# Bromine and Its Preparations

**Published:** 1846-12

**Authors:** 


*Bromine and its Preparations.*—The very high price which iodine has attained within the last twelve months, has rendered it very desirable that a substitute should, if possible be obtained for this medicine, which is at present so extensively employed. Bromine and its preparations have been shown by the experiments of Majendie, Barthez, Brame, and others, to possess therapeutical properties as nearly as possible identical with those of iodine and the iodides. The scarcity, however, of bromine, and, consequently, its commercial value, has hitherto prevented its general employment as a remedial agent; but the recent discovery of it in large quantities in America has recalled attention to this substance as a substitute for iodine. Mr. O'Reilly, of this city, while lately in the neighborhood of New York, having had his attention called to the peculiar properties of the mother waters of many brine springs in the United States—the result of their evaporation for procuring common salt, found by experiment that they contained bromine in large quantities—nine drachms in every gallon. Having procured a large amount of bromine from this source, he has brought a hundred pounds weight of it home, and states that he can obtain an almost unlimited supply of it; the price at which it is now sold in

Dublin is eighteen pence an ounce, while the present price of iodine is three shillings and sixpence an ounce. These circumstances have induced us to include in our retrospect a short account of the doses and mode of administration of bromine and its preparations.

The forms in which it has been used on the Continent are, in the simple state much diluted, and combined in the form of bromides with potassium, barium, calcium, iron, and mercury. These preparations are made by processes exactly similar to those used for procuring the corresponding combinations of iodine. As a substitute for the tincture of iodine, M. Pourche has employed the following solution: bromine, one part; distilled water, forty parts; dose, from five to six drops in some aqueous vehicle, three or four times daily. For external use he employs a solution four times as strong as this. The bromide of potassium is very soluble in water, sparingly soluble in alcohol: the dose of it is from four to eight grains three times a day: to prepare an ointment from it, four parts are rubbed up with thirty-parts of lard; and if a stronger ointment, or one resembling the compound iodine ointment, be wished for, six drops of bromide are added to this. The bromide of barium is also soluble in water; the dose of it is from one to five grains, three times a day; the ointment is prepared by combining it in the proportion of one part to ten of lard. The bromide of calcium is prescribed in the form of pill made with the conserve of roses; the dose of it is from three to ten grains. The bromide of iron is a brick-red deliquescent salt, very soluble in water; it is not so easily decomposed as the iodide of iron, and is given usually in the form of pill made with conserve of roses and gum arabic; the dose of it is from one to three grains: it has been employed externally, also in the form of ointment, prepared with one part of the bromide to fifteen of lard. Two bromides of mercury have been used; the first a sub-bromide, is a white insoluble powder: the dose of it is one to two grains daily; the second, a bromide, is fusible and volatile, and soluble both in water and alcohol: its dose is one-sixteenth of a grain, gradually increased to one-fourth of a grain, daily.

All the preparations of bromine may be readily known from those of iodine by their not disengaging violet-colored vapors when concentrated sulphuric acid is poured on them. In France, bromide of potassium has been of late fraudulently sold for iodide of potassium, in consequence of the high price of the latter; a sophistication of but little importance, if, as we are inclined to believe, the medicinal action of both be identical.—*Dublin Quarterly Journal of Med. Science.—Ibid.*

